# Adjusted COVID-19 booster schedules balance age-dependent differences in antibody titers benefitting risk populations

**DOI:** 10.3389/fragi.2022.1027885

**Published:** 2022-10-12

**Authors:** Lisa Müller, Marcel Andrée, Wiebke Moskorz, Ingo Drexler, Sandra Hauka, Johannes Ptok, Lara Walotka, Ramona Grothmann, Jonas Hillebrandt, Anastasia Ritchie, Laura Peter, Andreas Walker, Jörg Timm, Ortwin Adams, Heiner Schaal

**Affiliations:** ^1^ Institute of Virology, Medical Faculty, University Hospital Düsseldorf, Heinrich-Heine University Düsseldorf, Düsseldorf, Germany; ^2^ Department of Nephrology, Medical Faculty, University Hospital Düsseldorf, Heinrich-Heine University Düsseldorf, Düsseldorf, Germany

**Keywords:** SARS-CoV-2, COVID-19, vaccination, humoral immune response, immunosenescence

## Abstract

We provide follow-up data on the humoral immune response after COVID-19 vaccinations of two distinct cohorts aged below 60 and over 80 years to screen for age-related differences in the longevity and magnitude of the induction of the antibody responses post booster-vaccinations. While anti-SARS-CoV-2 spike-specific IgG and neutralization capacity waned rapidly after the initial vaccination schedule, additional boosters highly benefitted the humoral immune responses especially in the elderly cohort, including the neutralization of Omikron variants. Thus, adjusted COVID-19 booster vaccination schedules are an appropriate tool to overcome limitations in the success of vaccinations.

## Introduction

From the onset of the SARS-CoV-2 pandemic in 2019, it quickly became clear that only prophylactic immunization offered a way out of this global health crisis (Dagan et al., 2021; Viana et al., 2021). In the following year 2020, several manufacturers received emergency use authorization for their vaccines against SARS-CoV-2. This included the novel class of mRNA vaccines, Comirnaty (BioNTech/Pfizer) and Spikevax/mRNA-1273 (Moderna) (Polack et al., 2020; Baden et al., 2021).

In Germany and other countries, immunization schedules were rolled out at the end of December 2020, prioritizing risk populations including immunocompromised and elderly individuals (Cylus et al., 2021). With mRNA vaccines being the first of their kind, vaccinated risk populations were in the focus of monitoring studies to evaluate the magnitude and quality of the immune response to these vaccines (Swift et al., 2021; Oliveira-Silva et al., 2022). While initially, both mRNA formulations were designed as “prime and boost” immunizations, several studies quickly pointed out the potential necessity of at least a third vaccination, especially for risk groups (Dagan et al., 2021; Wieske et al., 2022). This includes the elderly, who generally have a reduced or shorter-lived immune response due to their ageing immune system (Frasca et al., 2011). Immunosenescence has been shown to play an evident role in both, SARS-CoV-2 infection scenarios, where higher mortality and morbidity rates have been reported for elderly as well as in the magnitude of the antibody response post-vaccination (Bajaj et al., 2020; Brockman et al., 2022).

With the emergence of more variants of concern such the currently predominant Omikron variants which again caused a sharp surge in cases worldwide, the necessity of adjusted vaccination schedules became more evident. Therefore, less than a year after the first vaccination campaigns started, several countries began their additional booster campaigns. However, the combination of increasing case numbers and both, hetero- and homologous booster vaccinations, predominantly using mRNA vaccines, has led to a complex mixture of SARS-CoV-2-specific immunological profiles throughout the population.

Early in 2021, we performed a cohort study with two distinct age groups, vaccinees below 60 and above 80 years (Müller et al., 2021a). Here, we present a follow up of this vaccinee cohort. We revisited the study cohort half a year and 1 year after their initial prime and boost vaccination. We monitored the cohort for their total anti-SARS-CoV-2 spike-specific and nucleocapsid-specific immune response as well as the magnitude of the neutralizing antibody response. Now that Omikron is the most prevalent variant, we also included the neutralizing antibody response to the Omikron BA.1 and BA.5. The follow-up of this cohort provides an important snapshot of current immunological profiles.

## Materials and methods

### Participants and sample processing

Participants were volunteers from the SBK nursing home in Cologne, Germany who participated in both blood samplings for first part of the study (blood collection BC#1 and BC#2, analyzed in Müller et al., 2021) and in both follow-ups (blood collection BC#3 and BC#4) and gave informed consent (*n* = 84). They received their first vaccination at the end of December 2020 (27.12.2020–29.12.2020) and their second on 19 January 2021, both with the BioNTech/Pfizer vaccine. All individuals received their third vaccination (97.7% BioNTech, 2.3% Moderna) asynchronously between September and November 2021. Additionally, 39 (46.4%) of the participants received a fourth vaccination in early February 2022 (10.02.2022–14.02.2022, 100% BioNTech), 8 of the younger vaccinees, 31 of the elderly. Participants who reported an infection 6 months prior to the second follow-up (*n* = 20/84) were analyzed separately. This resulted in a final cohort of 64 vaccinated individuals (28 younger, 36 elderly). Blood samples for the 6-month post-vaccination control were collected on 29 July 2021. Blood samples for the 12-month post-vaccination visit were collected on 1 March 2022. Samples were aliquoted and stored at 4°C for direct use and −20°C for long term storage. Control samples were included in all assays. Cohort characteristics of the final study cohort can be found in Table 1.

**TABLE 1:** Cohort characteristics of the final study cohort.

	n	Male	Female	Age	Vaccination 3 (September–November 2021)	Vaccination 4 (10.02.2022–14.02.2022)
Overall	84	26	58	68.1 (24–100)	84 (97.7% BioNTech, 2.3% Moderna)	52 (100% BioNTech)
**Vaccinated**						
Overall	64	20	44	69.1 (29–100)	64	39
>80	36	12	24	88.1 (81–100)	36	31
<60	28	8	20	44.9 (29–60)	28	8
**Convalescent**						
Overall	20	6	14	64.3 (24–93)	20	13
>80	10	2	8	86.1 (81–93)	10	8
<60	10	4	6	42.6 (24–57)	10	5

### Assays

Blood Samples were tested for anti-SARS-CoV-2-spike antibodies using the commercially available Anti-SARS-CoV-2 QuantiVac Enzyme-Linked Immunosorbent Assay (ELISA) test system from Euroimmun and run on the Euroimmun Analyzer I-2P according to the manufacturer’s instructions. The anti-SARS-CoV-2-nucleocapsid IgG chemiluminescent microparticle immunoassay (CMIA) from Abbott was performed on an ARCHITECT i2000 SR.

A serial dilution endpoint neutralization test was performed with an infectious SARS-CoV-2 B.1 WT (EPI_ISL_425126), Omikron BA.1 (EPI_ISL_12813299.1) and BA.5 (EPI_ISL_14167576) isolate as previously described (Müller et al., 2021).

### Statistical analysis

Statistical analysis was performed using GraphPad Prism software Version 9.3.1.

## Results

**FIGURE 1:**
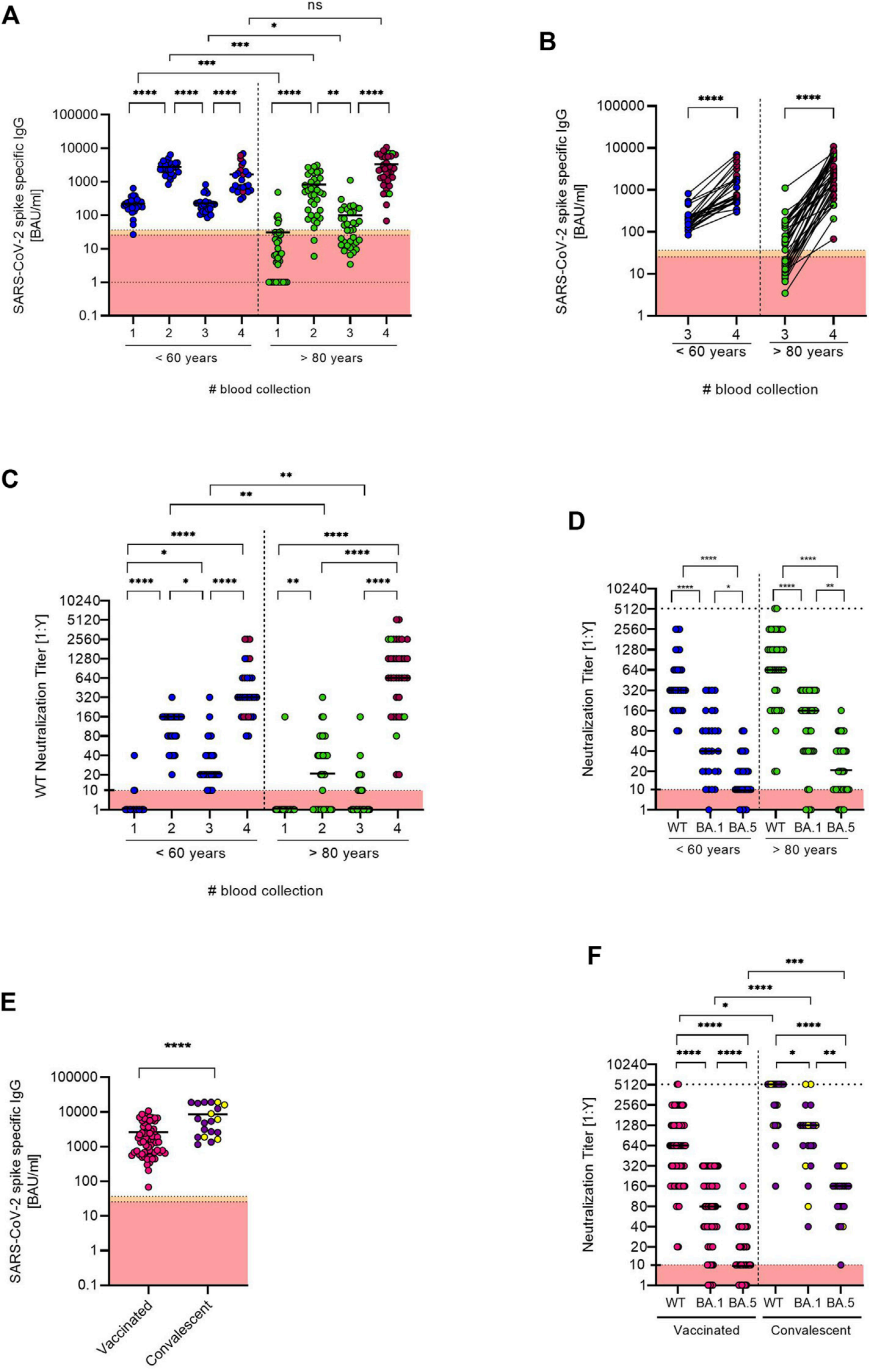
**(A)** Anti-SARS-CoV-2 spike specific antibody titers were determined for both age groups at each blood collection (BC#1 and BC#2 analyzed in Müller et al., 2021 [9]). Antibody titers below the detection limit were set to 1.0. Median titers are indicated as black bars. Individuals who received an additional booster vaccination before the fourth blood collection are indicated with red symbols. **(B)** Pairwise comparison of anti-SARS-CoV-2 spike IgG titers between younger and elderly vaccinees at BC#3 and BC#4. Individuals who received an additional booster vaccination before the fourth blood collection are indicated with red symbols. **(C)** Neutralization titers of the two age groups were measured at each blood collection. Individuals who received an additional booster vaccination before the fourth blood collection are indicated with red symbols. **(D)** Comparison of the neutralization titers against a B.1 WT strain and Omikron BA.1 and BA.5 isolates between the two age groups at the fourth blood collection. **(E)** Comparison of anti-SARS-CoV-2 spike IgG titers between vaccinated only and vaccinated convalescent individuals at the fourth blood collection. Convalescent individuals with an anti-SARS-CoV-2 nucleocapsid-specific IgG titer considered positive are marked in yellow. **(F)** Comparison of the neutralization titers against B.1 WT and BA.1 and BA.5 Omikron isolates of the vaccinated only and vaccinated convalescent cohort. All data sets were tested with the Shapiro-Wilk test for normality. As most data sets did not show normal Gaussian distribution, parametric tests were performed. Two unpaired data sets **(E)** were compared by two-tailed Mann Whitney test. Comparison of two paired data sets **(D,F)** were done by two-tailed Wilcoxon matched-pairs signed rank test. For the comparison of antibody titers at different time points **(A–C)**, tests within age groups were tested with two-tailed Friedman Test and comparison between age groups were tested with Kruskal Wallis Test (between), both followed by Dunn’s multiple comparison test. All tests were performed using GraphPad Prism software Version 9.3.1.

At the first follow-up blood collection (BC#3) 6 months after their first vaccination, the quantitative SARS-CoV-2 spike-specific IgG titers again differed significantly between the two groups (*p* = 0.0435). For the group of elderly vaccinees, the mean IgG titer was 99.98 BAU/ml, ranging from 3.45 to 1111.0 BAU/ml. In this group, 41.6% of the tested individuals had titers below cut-off (>35.2 BAU/ml). In the younger cohort, IgG titers ranged from 84.3 to 823.5 BAU/ml with a mean of 231.6 BAU/ml with no participants testing below cut-off (Figure 1A). At the second follow-up (BC#4) more than 1 year after the initial vaccination schedules, all participants had received a third vaccination. Furthermore, 86% (*n* = 31) of the elderly participants received a fourth vaccination, while only 28% (*n* = 8) of the younger participants received the additional booster. At this blood collection (BC#4), SARS-CoV-2 spike-specific IgG titers were comparable between the age groups. Interestingly, the mean SARS-CoV-2 spike-specific IgG titer in the group of elderly vaccinees was two-fold higher (3337 BAU/ml) than for younger vaccinees (1663 BAU/ml) and no participant tested below cut-off. However, titers ranged wider in the former group (68.0–10800 BAU/ml) than in the latter (300–6950 BAU/ml). The pairwise comparison of anti-SARS-CoV-2 spike IgG titers between younger and elderly vaccinees at BC#3 and BC#4 shows a general increase of titers, with those who received two additional vaccinations tending to have a higher overall titer (Figure 1B).

Furthermore, we compared the time-dependent progress of the neutralization capacity against a SARS-CoV-2 B.1 WT isolate between the age groups (Figure 1C). At the first follow-up (BC#3), the median neutralization titer (MNT) in the group of elderly participants drastically decreased to 0. After the elderly participants received their third and fourth vaccination (BC#4), however, the MNT for the group of elderly participants was 640. For the group of younger vaccinees, MNT were significantly higher compared to the elderly. At the first follow-up (BC#3), median neutralization capacity against the B.1 WT isolate in this group was decreased to 20. At the 1-year follow-up blood collection (BC#4), the MNT in the group of younger vaccinees was 320.

Measured at BC#4, the neutralization capacity determined by MNT was significantly decreased against both Omikron variants (BA.1 and BA.5) in both groups as compared to the B.1 WT. The MNT against Omikron BA.1 in the group of elderly participants was 160, against BA.5 it was decreased to 20 compared to 640 against the WT. Younger vaccinees showed an MNT of 40 against BA.1 and 10 against BA.5 compared to 320 against the WT isolate (Figure 1D).

Furthermore, we separately analyzed the group of vaccinated convalescent participants (*n* = 20) compared to the overall vaccinated cohort (*n* = 64). The 20 convalescent participants (10 younger and 10 older vaccinees), reported an infection confirmed by PCR within the past 6 months prior to the second follow-up blood collection, 9 were infected in a community outbreak in early February 2022. A comparison of overall anti-SARS-CoV-2 spike-specific IgG titers showed significantly higher titers (*p* < 0.0001) in the group of convalescent participants with a mean titer of 8560 BAU/ml compared to 2605 BAU/ml in the vaccinated cohort. It is of note that only 6 of 20 convalescent participants had anti-SARS-CoV-2 nucleocapsid-specific titers considered positive (Figure 1E).

In this cohort, we also analyzed neutralizing antibodies and compared the WT and Omikron titers against the vaccinated cohort. In line with overall anti-SARS-CoV-2 spike-specific IgG levels, the convalescent cohort displayed significantly higher neutralizing antibody levels against WT and both Omikron isolates. However, despite the majority being infected with Omikron during the community outbreak in February 2022, Omikron neutralization titers were still lower than WT titers (Figure 1F).

## Discussion

We present the follow-up analysis of the SARS-CoV-2-specific antibody response to the BioNTech/Pfizer prime/boost vaccination of a cohort consisting of two age groups half a year and 1 year after their first COVID-19-vaccination.

Both mRNA vaccinations that received emergency approval in late 2020 were initially designed as “prime/boost” vaccination schedules, however, longitudinal effects of the vaccinations were still to be determined. While it is evident that prophylactic immunizations decreased the pandemic burden and positively influenced the development of hospitalization and death rates (Moghadas et al., 2021), various studies pointed out potential limitations of the COVID-19-vaccinations in specific sub-cohorts. These include immunocompromised patients, in particular organ transplant recipients (Lee et al., 2022) as well as elderly where the reduced induction of the humoral immune response after vaccination can likely be attributed to the effect of immunosenescence. The impaired humoral immune response to immunizations has been described for various vaccines including hepatitis B, pneumococcal, and influenza vaccinations where strategies are in place to overcome limitations (Crooke et al., 2019; Schenkelberg, 2021). These include the use of certain adjuvants as well as higher dosages or adjusted vaccination schedules, routes which are all explored for COVID-19 vaccinations as well (Connors et al., 2021).

In this follow-up study, we observed a drastic decrease of the anti-SARS-CoV-2 spike-specific IgG titer at the first follow-up blood collection 6 months after the cohort received their first vaccination. Especially in the group of elderly participants, these effects were even more pronounced, with more than a third of the elderly vaccinees testing below cut-off. This is in line with other studies which report a rapid antibody waning after prime/boost regimens (Shrotri et al., 2021). It further underlines the necessity of strategies to overcome such limitations with additional boosters.

Thus, shortly after the first follow-up, study participants received an asynchronous third mRNA vaccination between September and November 2021, less than a year after their first vaccination. In early February 2022, Germany’s vaccination commission recommended another booster for risk groups, so the majority of elderly participants received a fourth vaccination before the second follow-up blood collection. This fully dispersed the age-dependent difference in mean IgG titers and neutralization capacity, although, as previously described, neutralization capacity against the Omikron BA.1 was drastically reduced, which was even more pronounced for BA.5 (Hachmann et al., 2022). Generally, long lasting spike-specific B-cells and T cell responses have been described to be found 8 months after the first vaccination dose in a two dose vaccination strategy even in the elderly (Parry et al., 2022). Nevertheless, an Omicron BA.1 outbreak in a nursing home in Kyoto City, Japan was accompanied by high mortality and morbidity in residents (median age 87 years old) that received their second (last) dose 7 month prior to the outbreak, underlining an insuffient immune response by a two dose vaccination strategy in the elderly (Matsumura et al., 2022). In line with that, several studies highlight the beneficial effects of a fourth vaccination in regards to elevated protection from severe illness, hospitalization and death and even short-lived protection from infection ans thus, proved highly beneficial for risk groups with participants aged over 60 (Arbel et al., 2022). Additionally, a recent study from Singapore found similar beneficial effect of a fourth vaccination compared to three doses only for individuals aged over 80 that got infected whilst the Omicron outbreak in early 2022 (Tan et al., 2022). As shown in large cohort studies with participants aged over 60, the fourth vaccination resulted in elevated protection from severe illness, hospitalization and death and even short-lived protection from infection and thus, proved highly beneficial for risk groups (Arbel et al., 2022; Bar-On et al., 2022).

In the group of convalescent individuals, the humoral immune response was significantly increased compared to the vaccinated cohort, which is in line with previous results (Müller et al., 2021b). While a high number of convalescents was infected during an Omikron BA.1 outbreak shortly before the second follow-up blood collection, both Omikron neutralization titers were lower than the neutralization titers against the B.1 WT, although neutralization titers against the WT were also significantly increased, suggesting a simultaneous induction of cross-reactive neutralizing antibodies against both variants (Garcia-Beltran et al., 2021). Furthermore, anti-SARS-CoV-2 nucleocapsid-specific antibodies traditionally used as serological marker for natural infections were only detected in the minority of convalescent individuals. With increasing break-through infections in vaccinated individuals and data from large-scale cohort studies that showed the rapid decline of nucleocapsid specific antibodies especially in vaccinees (Krutikov et al., 2022), it seems highly likely that this serological marker will become even more unreliable with the rise of more complex immunological profiles.

The asynchronous third vaccination as well as potentially unrecognized infections due to the lack of anti-SARS-CoV-2 nucleocapsid-specific antibodies are obvious limitations of this study. Furthermore, underlying health issue and medications can have a drastic effect on the humoral immune response and the resulting antibody titers. However, with our results, we would like to present a snapshot of a heterogenous population and therefore, did not stratify for such factors.

In conclusion we can show that in this cohort, the age-related differences in the humoral immune response have been balanced by an additional booster vaccination in the elderly cohort. Thus, adjusted booster schedules especially for risk groups are highly beneficial.

## Data Availability

The raw data supporting the conclusion of this article will be made available by the authors, without undue reservation.
